# Uncovering Essential Tremor Genetics: The Promise of Long-Read Sequencing

**DOI:** 10.3389/fneur.2022.821189

**Published:** 2022-03-23

**Authors:** Luca Marsili, Kevin R. Duque, Rachel L. Bode, Marcelo A. Kauffman, Alberto J. Espay

**Affiliations:** ^1^James J. and Joan A. Gardner Center for Parkinson's Disease and Movement Disorders, Department of Neurology, University of Cincinnati, Cincinnati, OH, United States; ^2^Consultorio y Laboratorio de Neurogenética, Centro Universitario de Neurología José María Ramos Mejía, Buenos Aires, Argentina

**Keywords:** long-read sequencing, whole-genome sequencing, genomics, tremor, movement disorders

## Abstract

Long-read sequencing (LRS) technologies have been recently introduced to overcome intrinsic limitations of widely-used next-generation sequencing (NGS) technologies, namely the sequencing limited to short-read fragments (150–300 base pairs). Since its introduction, LRS has permitted many successes in unraveling hidden mutational mechanisms. One area in clinical neurology in need of rethinking as it applies to genetic mechanisms is essential tremor (ET). This disorder, among the most common in neurology, is a syndrome often exhibiting an autosomal dominant pattern of inheritance whose large phenotypic spectrum suggest a multitude of genetic etiologies. Exome sequencing has revealed the genetic etiology only in rare ET families (*FUS, SORT1, SCN4A, NOS3, KCNS2, HAPLN4/BRAL2*, and *USP46*). We hypothesize that a reason for this shortcoming may be non-classical genetic mechanism(s) underpinning ET, among them trinucleotide, tetranucleotide, or pentanucleotide repeat disorders. In support of this hypothesis, trinucleotide (e.g., GGC repeats in *NOTCH2NLC*) and pentanucleotide repeat disorders (e.g., ATTTC repeats in *STARD7*) have been revealed as pathogenic in patients with a past history of what has come to be referred to as “ET plus,” bilateral hand tremor associated with epilepsy and/or leukoencephalopathy. A systematic review of LRS in neurodegenerative disorders showed that 10 of the 22 (45%) genetic etiologies ascertained by LRS include tremor in their phenotypic spectrum, suggesting that future clinical applications of LRS for tremor disorders may uncover genetic subtypes of familial ET that have eluded NGS, particularly those with associated leukoencephalopathy or family history of epilepsy. LRS provides a pathway for potentially uncovering novel genes and genetic mechanisms, helping narrow the large proportion of “idiopathic” ET.

## Introduction

Genetic factors contribute to various neurodegenerative diseases, as historically demonstrated by the study of selected families harboring specific pathogenic variants (1, 2). The development of next-generation sequencing (NGS) technologies during the last 15 years has accelerated the discovery of novel familial movement disorders and disease-causing variants in unusual sporadic phenotypes (2). However, despite the increase in genes and variants discovered in this group of disorders, routine NGS techniques have intrinsic limitations, mainly due to their ability to only sequence short-read length fragments (150–300 base pairs— bp) (3, 4) (Table 1). These limitations were among the motivations for the development of a new sequencing technology, the long-read sequencing (LRS).

**Table 1:** Main limitations of short-read next-generation sequencing (NGS) which may be overcome by long-read sequencing.

**DNA structure**	**Why short-read NGS misses it**
**Structural variants (SVs)**	Structural variants such as insertions, deletions, or inversions larger than 1 kilo-base (Kb) pair cannot be detected [76% improved detection of SVs with LRS when compared with short-read NGS (5, 6)]
**Copy number variants (CNVs)**	Genetic traits involving the number of copies of a particular gene present in the genome of an individual are mostly undetectable
**Widespread repeats**	Repetitive sequences are poorly or not detected
**Segmental duplications**	Sequences with numerous homologous elements are poorly or not detected

There are two main long-read platforms that have in common the ability to sequence in real-time single molecules of DNA, PacBio family (PacBio CCS) and Oxford Nanopore Technologies (ONT). Unlike the short-read NGS (mainly provided by Illumina platforms), LRS allows longer reads (over 10 kilo-base pair –Kbp in one single read) with high accuracy, thereby reducing the number of individual reads needed for complete coverage of individual genomic regions (4, 7, 8). Of note, long reads are single DNA molecules, and ONT reads can be ultra-long, even Mb-sized (9). LRS platforms offer the possibility of evaluating long reads of several kilobases, including highly repetitive regions, and significantly reduce GC errors. These advantages and new variant calling algorithms [namely, the algorithms used for the identification of variants from sequence data (10)] have recently allowed the detection and validation of variants which only a few years ago had eluded detection by short-read sequencing (8).

LRS technologies are currently used in several fields of neurology and have been the source of newly discovered pathogenic mutational mechanisms (4). Here we systematically review the main accomplishments of LRS technologies in the field of neurodegenerative disorders and focus on their potential utility on the syndrome of essential tremor (ET), where we envision an opportunity for their clinical application. In fact, the genetic etiology of some ET subtypes might be uncovered with LRS, particularly when other “pluses,” or atypical features, may be present, such as family history of epilepsy or associated white matter abnormalities on imaging. We will also discuss the main challenges of LRS technologies, and provide an overview of future applications of these exciting new methodologies in research. To facilitate understanding, we have created a glossary of key terms.

## Long-Read Sequencing: From Limitations to Opportunities

### Limitations of Next-Generation Sequencing

It has become clear that the human genome variability is caused by structural variations more commonly than previously thought (11). Structural variants (SVs) are currently defined as insertions, deletions, or inversions larger than 1 Kbp (This cutoff is arbitrary, and others may refer to mutations >50 bp as SVs) (12). Copy number variants (CNVs) refer to the number of copies of a particular gene present in the genome of an individual (13). The expanded use of sequencing techniques in clinical practice has increased, at least in part, the spectrum of both SVs and CNVs routinely identified. However, the information amenable to be obtained by classical short reads NGS platforms is still not sensitive enough for SVs and CNVs detection (11). Moreover, a clear limitation of short-read sequencing appears when regions of the genome harboring SVs, GC-rich regions, and repetitive sequences or sequences with many homologous elements (widespread repeats and segmental duplications) (3, 7) need to be mapped to reference assemblies (Table 1). Repetitive sequences of DNA are highly-prevalent across different species ranging from bacteria to humans, and they account for about 50% of the human genome (3).

### Repeats Constitute a Huge Challenge for Sequence Alignment and Assembly Preparations

**Figure 1:**
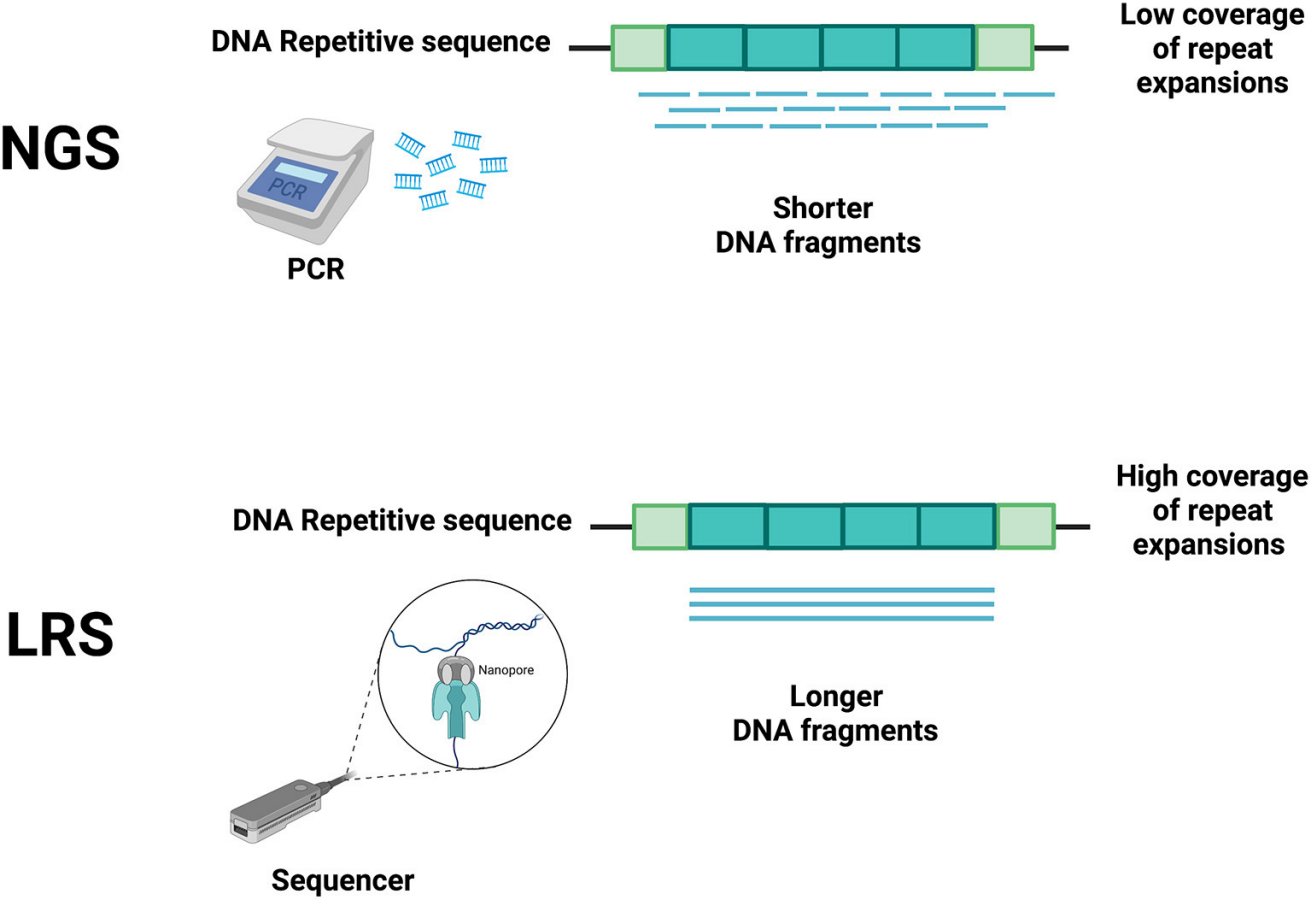
Main differences between short-read next-generation sequencing (NGS) and long-read sequencing (LRS) technologies. Short-read NGS (upper panel) uses PCR to obtain short DNA fragments that do not accurately cover the whole repeated DNA sequence, thus allowing many interpretation errors (e.g., including edge of repeat and flanking regions, spanning boundaries of repeats). The differing technology of LRS (lower panel) does not require PCR, allowing a more accurate coverage of the repeated DNA sequence. Created with BioRender.com.

NGS techniques, due to their short read lengths, have the disadvantage of making these problems harder to solve (3). Hence, in the last 20 years, several technological developments have allowed increasing both the speed and the length of sequencing reads with the goal of reducing cycle times, accelerating sequence assembly, and enabling the accurate sequencing of repeat-rich areas of the genome (14–16). These discoveries have allowed a fast and relatively low-cost genome sequencing (16), facilitating the development of LRS, where the sequencing process is in real-time and polymerase chain reaction (PCR) amplification is no longer necessary (Figure 1). With the reading of longer fragments, LRS raises the accuracy in read mapping (3, 4) enhacing the odds for sequencing-generated discoveries where short read sequencing has previously failed (4, 7, 8, 17, 18).

### Long-Read Sequencing in Neurodegenerative Disorders

LRS has been used to successfully sequence patients with diseases associated with repeat expansions. It permitted the identification of pathogenic trinucleotide and pentanucleotide repeat expansion disorders, as the examples below illustrate.

### The Example of Spinocerebellar Ataxia Type 10

Spinocerebellar ataxias (SCAs) are a heterogeneous group of conditions associated with ataxia (19). Some SCAs are caused by exonic CAG trinucleotide repeat expansions in selected coding regions, translating into long polyglutamine chains (20). However, non-coding repeat expansions and other conventional mutations may also cause other types of SCAs (4). As an example, SCA10 is caused by a noncoding ATTCT pentanucleotide repeat expansion located in intron 9 of the *ATXN10* gene (21). SCA10 shows a wide range of phenotypic variability, which may include epileptic seizures (21).

From a technical standpoint, previous NGS studies of repeat expansions were based on amplicon sequencing. For disorders caused by large expansions such as SCA10, this approach is not feasible (22). Therefore, for an optimal analysis of these large intronic repetitions, it has been proposed to sequence DNA without using any preceding PCR amplification (23). For this purpose, a specific CRISPR/Cas9-based amplification-free target enrichment method (also known as No-Amp targeted sequencing) was created (24). Through the combination of LRS technologies matched with the no-Amp approach, it has become possible to identify interruption motifs, potentially acting as phenotypic modifiers, within the tandem repeat expansion locus in *ATXN10* in patients presenting with variable phenotypes not always in the spectrum of SCA10, including a Parkinson's disease (PD) phenotype (25, 26). The hypothesis is that the interruptions present in the SCA10 repeat expansion may serve as a “phenotypic modifier” of the genotype-phenotype correlation between *ATXN10* and parkinsonism (4, 26).

Targeted-LRS (T-LRS) can be obtained not only with the No-Amp sequencing, but also using PCR enrichment. The T-LRS allows to use LRS for selected, targeted genomic regions with computational method, thus permitting to select and sequence native DNA (18). This approach is called adaptive sampling T-LRS, and permits to accept or reject the DNA fragments for sequencing, based on pre-set target sequences with the advantage to be modified in real time (27, 28) (further in *Discussion*).

### The Example of Huntington Disease

Huntington disease (HD) is caused by a CAG repeat expansion in the *HTT* gene located on chromosome 4 (29). The number of repeats inversely correlate with the disease severity and the age at onset (30). Hence, the precise calculation of the number of repeats has crucial diagnostic and prognostic values. To date, due to the intrinsic limitation of the PCR stutter and high GC content, NGS techniques are limited in the diagnostic accuracy of HD. For this purpose, the No-Amp targeted sequencing has been used to overcome the difficulties due to PCR stutter and to the high GC content, thus permitting a detailed sequence information and the assessment of somatic variability of repeated elements without the confounding errors related to the PCR stutter (7). In addition, LRS demonstrated the feasibility of rapidly genotyping and haplotype phasing to differentiate the genetic variability inherited either from paternal or maternal origins without familial genotype/haplotype data (31). In HD, this has led to determine the allele bearing both the pathogenic CAG expansion and SNPs 165 kb downstream, essential for personalized therapies.

### The Example of Amyotrophic Lateral Sclerosis/Frontotemporal Dementia

A similar approach has been in amyotrophic lateral sclerosis (ALS)/frontotemporal dementia (FTD) associated with *C9ORF72* gene variants (23, 32). ALS and FTD are considered a spectrum of disorders that may share some common genetic bases (27). In particular, the documentation of an expanded hexanucleotide repeat in the noncoding region of *C9ORF72* gene on chromosome 9, has linked ALS with FTD genetic variants (33). Again, due to its high length and GC content, the hexanucleotide repeat expansion was difficult to sequence (32, 34, 35), but LRS technologies made it possible (4, 32).

### The Example of Genetic Parkinsonism

LRS technologies have been useful in investigating two genetically-determined parkinsonisms, namely X-linked dystonia-parkinsonism (XDP) and GBA-related parkinsonism. XDP is a monogenic movement disorder presenting with both dystonia and parkinsonian features (36). The underlying genetic mechanisms of XDP have been thoroughly investigated using LRS techniques which, initially, narrowed the causal mutation to the *TAF1* gene locus, thus hypothesizing an alternative splicing defect mechanism (Using PacBio, *see also Section 4*) (37). Later, thanks to the Nanopore LRS technologies (*See also Section 4*), it has been demonstrated that alternative splicing of *TAF1* is not the molecular basis for this disease, thus marking a paradigm shift in the understanding of this condition and suggesting that the decrease in *TAF1*-messenger RNA is implicated in XDP pathogenesis (38). In *GBA*-related parkinsonism, heterozygous mutations in the *GBA* gene are considered strong risk factors for this condition. However the analysis of *GBA* is complicated by the nearby pseudogene which shares 96% exonic sequence homology to the *GBA* coding region (39). As noted above, LRS technologies have been useful in detecting disease-causing intronic and exonic variants with good haplotype phasing information (39, 40).

### Systematic Review of the Literature on Long-Read Sequencing in Neurodegenerative Disorders

To better understand the impact of LRS technologies in neurodegenerative research, we performed a systematic review of the LRS literature focusing on new mutations discovered by LRS. We also searched for studies about LRS currently active or soon-to-be active in neurodegenerative diseases. We found 59 articles in PubMed and Cochrane, and only one record in ClinicalTrials.gov of a study not yet recruiting, aimed to analyze through LRS already existing biological samples from patients with specific mutations for neurodegenerative diseases. The main results of our systematic search (Table 2, Supplementary File) demonstrates the increasing impact LRS is making in the discovery of new pathogenic variants in previously unidentified genes and novel mutational mechanisms, especially those comprising repetitive and large structural variation. Importantly, 10 of the 22 (45%) genetic etiologies ascertained by LRS included tremor in their phenotypic spectrum.

**Table 2:** Mutations previously hidden from standard genetic techniques but resolved after long-read sequencing^a^.

**Gene locus (PubMed and cochrane identifier)**	**Disorder**	**Mutation**	**Atypical features**
*ATXN10*	PD	ATTCC repeat expansion without ATTCC repeat interruptions (26)	Young-onset parkinsonism, normal brief smell identification test, mild cerebellar atrophy
	SCA10	ATTCT repeat expansion with ATTCC, ATCCT and ATCCC very-long repeat interruptions (25, 26, 41)	Focal motor seizures with impaired awareness 10–30 years after ataxia onset in patients with ATTCC repeat interruptions
*CLN6*	PME	12.4 Kb deletion within a GC-rich region, and altering the gene's initiation codon (42)	Severe phenotype with regression, progressive diffuse atrophy and thin corpus callosum
*CR1*	AD	Ten-nucleotide frameshift deletion in one of exons 10, 18 or 26 (43)	Two patients were first-degree relatives
*C9ORF72*	ALS/FTD	GGGGCC repeat expansion (32, 44–47)	No
*DMD*	BMD phenotype	Acquisition of a new exon from the retrotransposable element LINE-1 (48)	No
	DMD phenotype	Complex structural variants: 7 Kb to 0.9 Mb inversions flanked by 0.1–3.8 Kb deletion-insertion rearrangements (49, 50). SVs of breakpoint sequences with repetitive regions in intron 44 (51)	No
*DMPK*	DM1	CTG repeat expansion stabilization/contraction associated with de novo CCG repeat interruptions (52–54)	Milder symptoms in patients with CCG repeat interruptions
*DPP6*	AD	Paracentric inversion of 4 Mb with the distal breakpoint in intron 1 of *DPP6*, disrupting its coding sequence (55)	Family with autosomal-dominant early-onset AD and with segregation of the mutation haplotype
*DUX4*	FSHD	Contraction of the subtelomeric macrosatellite repeat D4Z4 (56, 57)	No
*FMR1*	FXS/FXTAS	CGG repeat expansion with and without AGG repeat interruptions (58–61). Different *FMR1* mRNA isoforms expressed (62)	Women with 60–85 CGG repeat expansions and higher AGG repeat interruptions were associated with to fewer chances of having children with FXS (expansion stabilization/contraction)
*GBA*	PD	55-bp exonic deletion with a pathogenic p.D448H missense mutation (39)	Not specified
*GIPC1*	OPDM2	CGG repeat expansion (63, 64)^a^	No
*LRP12*	OPDM1	CGG repeat expansion (64)	No
*MARCH6*	FAME3	TTTCA/TTTTA repeat expansion (65)	No
*MIR222*	X-linked intellectual disability	Two consecutive (CT)_n_ and (GT)_n_ repeat expansion (XLID25 repeat) (66)	No
*NOTCH2NLC*	ET	GGC repeat expansion (67, 68)	More severe phenotype, genetic anticipation, and few patients with other symptoms^b^
	Hereditary distal motor neuropathy and rimmed vacuolar myopathy	GGC repeat expansions (69)	Rest and postural tremor in both hands, mildly high signal of the splenium of corpus callosum
	MSA	GGC repeat expansion (70)	Longer disease duration (>8 years), prominent cognitive impairment, mild-to-moderate cortical atrophy and mild white matter lesions
	NIID	GGC repeat expansion with and without GGA repeat interruptions (71–74). Also, CGG repeat expansion (75)	No
	OPDM3	GGC repeat expansions (76)	White matter hiperintensities
	PD	GGC repeat expansion without GAA repeat interruptions but with or without AGC repeat interruptions (77)	Typical PD responsive to small dosages of levodopa (150–300 mg) for 10–20 years without motor fluctuations, and with mild cerebral atrophy
	Recurrent encephalopathy, postural tremor and parkinsonism	GGC repeat expansion (68)	Migraine, reversible focal neurological deficits, status epilepticus. DaTScan normal. Multifocal cortical and subcortical signal abnormalities without hyperintensity in corticomedullary junction in DWI.
*RAPGEF2*	FAME7	TTTTA/TTTCA/TTTTA repeat expansion (78)	No
*RFC1*	CANVAS	AAGGG repeat insertion (79)	Auditory hallucinations
*SAMD12*	FAME1	Three possible configurations of repeat expansions: TTTTA/TTTCA, TTTTA/TTTCA/TTTTA and TTTTA/TTTGA/TTTCA (42, 65, 78, 80–85)	No
*SAMD9L*	APS	Two distant, paternally inherited missense mutations p.R359Q and p.Y1118C (86)	Severe immune dysregulation, raised CSF protein levels, cerebellar atrophy and extensive supratentorial gray and white matter signal changes
*STARD7*	FAME2	ATTTC repeat expansion (87)	No
*TAF1*	X-linked dystonia-parkinsonism	CCCTCT repeat expansion within the SINE-VNTR-*Alu* retrotransposon insertion (88)	No
*TNRC6A*	FAME6	TTTTA/TTTCA/TTTTA repeat expansion (78)	No
*YEATS2*	FAME4	TTTCA/TTTTA repeat expansion (89)	No
**Future studies**			
**ClinicalTrials.gov** **Identifier**	**Study population**	**Gene loci**	**Objectives**
NCT04621422^c^	Patients carrying mutations on selected genes	*FMR1, DMPK, ZNF9, SCA2, JPH3, HD, FXN, C9ORF72, RFC1*	Compare repeat amplification results between Next Generation Oxford sequencing and reference PCR techniques

a *Reference (63) reports the repeat expansion “GGC” which should have been named “CGG,” applying the HGVS 3' rule*.

b *Cognitive impairment, frequent urination, hypermyotonia, ataxia, pyramidal signs, peripheral neuropathy or paroxysmal loss of consciousness*.

c *Study not yet recruiting disclosed to analyze already existing biological collections from patients carrying the specified mutations*.

*AD, Alzheimer's Disease; ALS/FTD, amyotrophic lateral sclerosis/frontotemporal lobar degeneration; APS, Ataxia-pancytopenia syndrome; BMD, Becker muscular dystrophy; CANVAS, cerebellar ataxia with neuropathy and bilateral vestibular areflexia syndrome; CSF, cerebrospinal fluid; DMD, Duchenne muscular dystrophy; DM1, myotonic dystrophy type-1; ET, essential tremor; FAME1, familial adult myoclonic epilepsy type 1; FSHD, facioscapulohumeral muscular dystrophy; FXS, Fragile X syndrome; FXTAS, fragile X tremor/ataxia syndrome; GD, Gaucher's disease; HD, Huntington Disease; MSA, multiple system atrophy; NIID, neuronal intranuclear inclusion disease; OPDM1, oculopharyngodistal myopathy type 1; PD, Parkinson's disease; PME, progressive myoclonus epilepsy; SCA10, spinocerebellar ataxia type 10; SVs, structural variants*.

## Long-Read Sequencing in Tremor-Related Disorders

### Long-Read Sequencing in Fragile X-Associated Tremor/Ataxia Syndrome

Some of the positive results of LRS technologies in the field of neurology have been accomplished on tremor-related disorders. Historically, the first application of LRS technologies in neurodegenerative disorders was on fragile X-associated tremor/ataxia syndrome (FXTAS) (58). FXTAS and fragile X syndrome (FXS) are both associated with a CGG trinucleotide repeat expansion on the *FMR1* gene located on the X chromosome (90). Abnormal repeats in FMR1 in the so-called premutation range of 55–200 repeats express as FXTAS whereas more than 200 repeats manifests as FXS (4), markedly reducing the expression of *FMR1*. Despite the shared genetic background, these two conditions have discrepant phenotypes. FXTAS patients develop prominent tremor, ataxia, cognitive impairment, and peripheral neuropathy (with many patients carrying a diagnosis of ET or “ET plus”); instead, FXS patients mostly develop cognitive impairment (91). However, the premutation range of trinucleotide repeats is unstable between generations. With subsequent expansions, it may reach a full mutation, particularly during maternal transmission (92). Interestingly, LRS technologies have shown that the presence of specific interruption sequences (AGG) into the trinucleotide repeats may reduce this instability (92). Therefore, LRS technologies accurately assess the number of repetitions and the presence of interruption sequences, thus allowing a more precise estimation of expansion risk and opening the possibility of preimplantation genetic diagnosis (93, 94). This level of information only obtainable by LRS greatly inform early diagnosis and family counseling in these two X-linked conditions (4).

### Long-Read Sequencing in Cortical Tremor—Familial Adult Myoclonic Epilepsies

Another interesting application of LRS technologies in the field of tremor-related disorders is on the familial adult myoclonic epilepsies (FAMEs) (75). FAME, also known as familial cortical myoclonic tremor with epilepsy (FCMTE) in Asia, belongs to the spectrum of the so-called benign adult familial myoclonic epilepsy (BAFME) (4, 75). These conditions are characterized by cortical tremor (phenomenologically tremor, electrophysiologically rhythmic myoclonus) and infrequent seizures with benign progression that segregate under an autosomal dominant familiar inheritance pattern (95, 96). As a result, many of these patients were considered within the spectrum of ET before their genetic etiology was unraveled (97). Although Mori et al. (98) mapped the disease locus to chromosome 8, through linkage analysis in 2011, no causative mutations were described until 2018 (75). Ishiura et al. (75) identified a pentanucleotide repeat expansion and some extra repeat sequences in a specific intron of the *SAMD12* gene in a pool of Japanese families with BAFME1, using LRS technologies. Later, this same pattern was confirmed in a large family of Chinese patients using LRS technologies, thus achieving two important results: 1) The confirmation of previously published results on Japanese pedigrees; and 2) The confirmation that LRS is effective for diagnosing genetically determined diseases, particularly when NGS-based technologies have failed to identify a causative mutation (80). Interestingly, other pentanucleotide repeats were also found in genes different from the *SAMD12*, such as *TNRC6* (BAFME6) and *RAPGEF2* (BAFME7) (75). Subsequently, intronic repeat expansions have been found in the *STARD7* (FAME2) and *MARCH6* (FAME3) genes (although using short-read NGS and not LRS technologies) (65, 87). Finally, intronic expansions in *YEATS2* were found as causal in BAFME4 (4, 89) (Table 2). In conclusion, similar tremor phenotypes have been caused by non-coding repeats, suggesting that in this class of disorders (BAFME/FAME/FCMTE) the presence of large repeats may underpin its pathogenesis to a greater extent than a single genomic locus *per se*.

### Long-Read Sequencing in Essential Tremor

In the field of ET, Sun and colleagues (67) recently demonstrated using a cohort of 197 participants that a GGC repeat expansion in the *NOTCH2NLC* gene can manifest as ET (5.8% of cases studied). They also found that the abnormal repeat expansion correlated with the severity of the phenotype and with anticipation in subsequent generations (67). Of interest, *NOTCH2NLC* has also been previously identified as the same gene causing neuronal intranuclear inclusion disease (NIID) (75), a condition characterized by peripheral neuropathy and cognitive impairment, phenotypically unrelated to ET. Furthermore, GGC repeat expansions (>79 repeats) in the *NOTCH2NLC* gene have been described in patients classified as “benign PD” (e.g., treated with low levodopa doses), and no other clinical or imaging features of NIID, after several years of follow-up (77). Taken together, these results suggest that there are subsets of patients affected with tremor within the “ET,” “PD,” and even “dystonic tremor” (DT) spectrum, for which LRS may identify causative loci and pathogenic mechanisms. Of note, *NOTCH2NLC* is highly homologous to other four paralogs of the human genome, making it extremely difficult to obtain its proper sequence when short-read NGS technologies are used.

*What is missing in the field of familial essential tremor?* To date, the definition and classification of tremor-related disorders is based on their phenotypic manifestations (99). ET is a syndrome, often inherited in an autosomal dominant pattern (100, 101). Due to discrepancies between studies, the estimated rate of inheritance is highly variable, ranging from 20% to 90% (101). Rare genes have been found in selected ET families by exome sequencing (e.g., *FUS, SORT1, SCN4A, NOS3, KCNS2, HAPLN4/BRAL2, USP46*) (101). If classical genetic mechanisms were at play, not only would have other families with these genetic etiologies would have been reported but other genes might have been identified. Based on the recent successes of LRS technologies, and in line with precision medicine principles, a new molecular approach is required (102–105). Conditions traditionally grouped under such clinical categories as “ET,” “ET plus tremor,” “orthostatic tremor” (OT), “pseudo-OT,” and “indeterminate tremor” could be phenotypic expressions of several genetic etiologies (106). This hypothesis is supported by the abnormalities recently discovered in genes associated with “ET/ET plus” conditions, such as trinucleotide (e.g., *NOTCH2NLC, FMR1*) and pentanucleotide (e.g., ATTTC repeats) repeat disorders (100, 102, 107) (Table 2). For this purpose, based also on the results of previous studies conducted in patients with FXTAS, FAME, and ET, we believe it is recommended to apply LRS to cohorts of patients with the phenotype of “ET”, potentially including those with familial bilateral hand tremor and any associated conditions, particularly epilepsy, or even minor white matter abnormalities changes. This approach is expected to reveal the genetic underpinnings of a certain subset of individuals currently classified within the broad “ET” spectrum. In fact, familial ET-like disorders have classically been described as segregating in an autosomal dominant fashion, yet only repeat expansion mutations (e.g., *NOTCH2NLC, FMR1*, ATTTC repeats) have been ascertained as pathogenic. We hypothesize that a reason for this glaring shortcoming may be the non-classical nature of the genetic mechanism(s) underpinning ET, among them trinucleotide, tetranucleotide, or pentanucleotide repeat disorders.

## Technical Challenges

Studying ET and other tremor-related disorders with a LRS-based approach will come with technical and methodological challenges. A first consideration entails the methodological strategy to adopt for this purpose. A possible solution could be to perform a genetic linkage analysis using microsatellite markers (80) or single nucleotide polymorphisms (SNPs) (108). Afterward, NGS-based technologies, namely whole exome sequencing (WES) and whole genome sequencing (WGS) can be used to identify the disease locus of interest (80). Finally, even if a low coverage (~10× -15× ) might suffice for studies that combine both technologies, a higher coverage is desirable with both PacBio CCS and ONT platforms to identify causative mutations (80). After that, repeat-primed PCR can be then applied for the possible validation and confirmation of the identified repeat expansions and rapid screening of other subjects at risk (80). We know that the LRS technologies are accurate in providing coverage for the most difficult regions of the genome (Table 1). However, there are sequencing challenges to be overcome by LRS technologies, including the analysis of telomeric regions, due to their high variability and number of masked satellites, are poorly covered by all long- and short-read technologies (109). Despite a great improvement in the last years, ONT platforms seem still behind other platforms in terms of accuracy of sequencing and coverage (109) although this is rapidly changing because of continuous updates in chemistry and bioinformatic analysis of this platform (9) (Table 3). PacBio CCS platforms are also more accurate for searching specific interruption sequences (e.g., AGG) as demonstrated in FXTAS (92). In particular, the circular consensus sequencing (CCS) protocol allows long, high-fidelity reads with high accuracy (113). Hence, PacBio CCS platforms allow the utilization of smaller fragment sizes, with a global quality higher than Illumina (109). To complicate things further, improved analysis of Illumina short read data is now possible thanks to new algorithms like the ExpansionHunter and GangSTR (68, 114, 115). To the best of our knowledge, a systematic comparison of these short-read analyses to LRS is not available. However, the big advantage of ONT platforms is the lower and more competitive cost for their service, when compared to PacBio CCS platforms. Also, it is estimated that within a year, ONT platforms will significantly improve their accuracy and quality. This aspect should be considered when envisioning long-term research efforts in which data analysis will be undertaken years after biospecimen collection (when improvements in both technologies will be available). Both ONT and PacBio CCS are more suitable for DNA extracted from blood instead of saliva or other tissues, as compared with short-read NGS (we have personally observed that compared to blood-isolated DNA, saliva-isolated DNA shows lower yields and lower molecular weight, resulting in much smaller DNA fragment sizes), another aspect to consider when planning a study. The final challenge is related to the fact that both LRS technologies are less accurate when the source material come from shorter DNA samples previously stored for other purposes, because of the need of a dedicated extraction of the DNA in bigger fragment sizes.

**Table 3:** Main features of the two long-read sequencing platforms: PacBio CCS and ONT.

**Features**	**PacBio CCS**	**Oxford Nanopore Technologies (ONT)**
Read lenght	> 10 Kbp	Up to 1Mbp
Pitfalls	High costs	Relatively high raw read error rates (7%, but reduced to 1.1% when applying isONcorrect); but lower costs
^*^Estimated costs per starter pack	~ $350,000 (PacBio Sequel)	Variable from $1,000 (MinION), to $50,000 (GridION) or $200,000 (PromethION)
Accuracy	> 99%	98%
Estimate of intraplatform reproducibility	Good	Unknown
Limitations	Low coverage of masked satellite regions	

*CCS, circular consensus sequence; Bp, base pair; isONcorrect is a novel computational method to correct errors in ONT cDNA sequencing data. Information retrieved from (109, 110)*.

* *Rough estimate; costs are highly variable depending on the specific needs. Data retrieved from (111, 112)*.

## Discussion

In the present review we have summarized the novel features of LRS genetic technologies, compared to short-read NGS as applied the field of ET and “ET plus.” The last decade has been a prolific era for the discovery of several genes (and related nucleotide repeat expansions) associated with tremor-related disorders and with other ostensibly unrelated phenotypes. Our systematic review confirmed the growth in new genes and genetic mechanisms discovered by LRS technologies, suggesting an underappreciated potential. Also, LRS technologies have revealed new insights about the relationship between coding and non-coding regions of our genome, thus improving the discovery of novel disease-causing variants. The discovery of these new variants must go in parallel with the development of the computational frameworks, thus bridging the gap between frontline research and practical efforts. In fact, the output of LRS-based studies clearly show that analysis of non-coding region is increasingly important in the regular workflows of clinicians determining the best approach for selected patients with suspected neurogenetic disorders (2).

The systematic application of LRS technologies in the field of tremor-related disorders will allow to determine the extent to which selected phenotypes are associated with repeat expansions. There is a high probability that the systematic application of LRS and WGS technologies will uncover novel repeat expansion disorders as potentially etiologic of “ET plus” and other tremor-related disorders. In the near future, LRS will be the first tier test for many tremor-related disorders and a serious alternative to short-read NGS (18).

The potential of LRS technologies may get us closer to elucidating the underlying genetic mechanisms of molecular subsets of ET on the road to precision medicine (102). While this approach can only be phenotype-driven at the outset, we envision a data driven, unbiased analytic approach as of higher hierarchical order in the future (116). This unique approach will allow a reverse biology-to-phenotype direction of research development in which clinical and imaging data are subordinate to genetic and biological signals of interest (116). A hypothesis free, causally- and data driven-based analyses, with an inclusive recruitment of patients with different phenotypes of neurodegenerative disorders, formed the foundations of the ongoing Cincinnati Cohort Biomarker Program (CCBP) study (116).

Promising results have come from a recent study conducted on over 3,000 inhabitants in Iceland, showing that LRS may improve the classification of SVs at the population level, when using a genome-wide non-targeted methodology (17). LRS can be useful for further large-scale efforts aimed at the exploration of different SVs unreachable with the NGS short reads sequencing approach (17). Another example was the findings of a common repeat expansion locus in *ATXN10*, associated with both ataxia (SCA10) and parkinsonism, two unrelated phenotypes (67, 77), suggesting that quite diverse phenotypes may share common genotypes. Another example of this successful strategy is that the GGC repeat expansion in the *NOTCH2NLC* gene may be associated with ET, “benign” idiopathic PD, and NIID, also three different phenotypes (67). Interestingly, genome-wide association studies have demonstrated an association between variants in the *LINGO1* gene and familial ET (117). Recently, a CNV (tandem duplication) affecting the *LINGO1* gene was found in a family with a predominantly dystonic tremor, using genome-wide SNP microarray analysis (118). Hence, *LINGO1* gene may be involved in both ET and dystonic tremor. However, the *LINGO1* risk variants are located in intronic regions, and the sequencing of *LINGO1* exons in ET patients have failed to identify any pathogenic variants (118).These studies did not include LRS; if used, LRS could have helped in solving the problem and, perhaps, in identifying the repeats in the intronic regions. In sum, we are confident that a LRS-based approach will allow the discovery of the biological and genetic underpinnings of several neurodegenerative conditions, at least in the field of tremor-related disorders.

**Figure 2:**
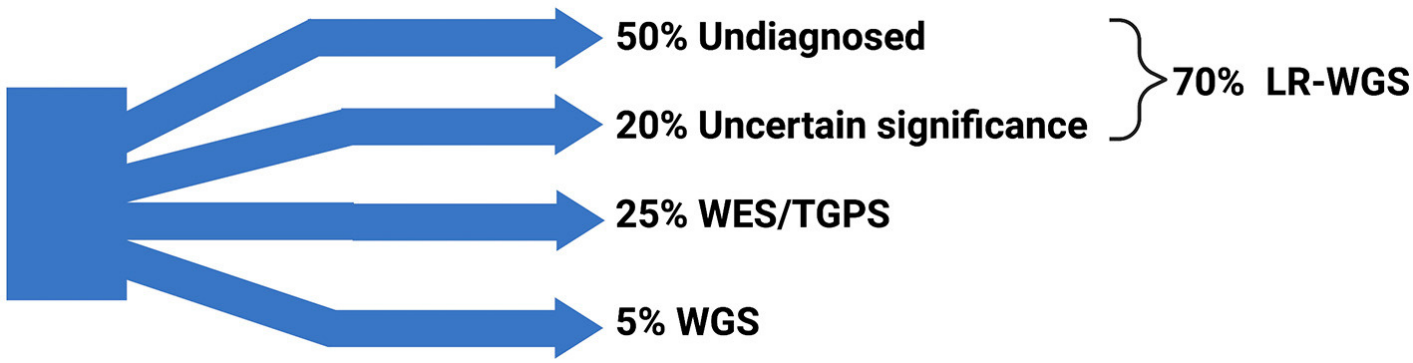
Traditional genetic workup and possibilities derived from long-read sequencing. The traditional (current) genetic workup is not diagnostic in about 50% of cases; other 20% of unresolved cases are represented by variants of uncertain significance. LRS (when coupled with whole genome sequencing— WGS, namely LR-WGS) has the potential to diagnose up to 70% of currently unresolved cases. The remaining 25% of cases are diagnosed using targeted genetic panel sequencing (TGPS) and whole exome sequencing (WES), and 5% of cases are diagnosed by WGS. Data derived from Salinas et al. (104) and Frésard et al. (119). Created with BioRender.com.

From a clinical standpoint, LRS is also useful to assign pathogenicity to a given detected variant especially when haplotypes can be assigned from long reads and not from short reads. We are aware that the genetic and phenotypic heterogeneity of neurogenetic diseases may force patients into a sort of “diagnostic odyssey” (104). A possible meaningful approach could be using a targeted gene panel sequencing combined with WES/WGS. From a previous combined experience in our centers (in Buenos Aires, Argentina and Cincinnati, USA), focusing on the usefulness of reinterpreting variants previously classified as of “uncertain significance,” about 30% of those variants were reclassified as pathogenic (104) using this approach. The use of NGS has allowed the identification of both germline and mosaic pathogenic variants, expanding the diagnostic yield (104). These results demonstrated the high clinical impact of periodic reanalyses of undetermined variants in clinical neurology. A major challenge is that the generation of LRS data for a thorough genome-wide analysis of these cases of “uncertain significance” is extremely expensive (18). For this purpose, the adaptive sampling T-LRS is a promising tool to broaden the clinical use of LRS with a cost comparable to the current one for the short-read WGS and with high sensitivity and specificity, although larger prospective studies are needed to confirm these results (18). We can assume that, in the future, the WG-LRS could easily replace almost every other genetic tests currently used in the clinical field (18) (Figure 2).

In sum, in the last decade the broad application of NGS and LRS technologies has helped interpret a large proportion of rare non-coding, missense, “uncertain significance,” and structural variants in the genome in several neurodegenerative diseases. We envision a great potential for the application of LRS in ET and “ET plus” disorders. This approach will help with the diagnosis, classification, and possibly also design of disease-specific treatment of patients with different neurogenetic neurodegenerative disorders. The development of LRS technologies are moving us closer to the desired reversed genotype-to-phenotype paradigm (2). This shift has the potential to usher a systems biology model of precision medicine for patients with neurodegenerative diseases.

## Author Contributions

LM: conception, organization, execution of the study design, writing of the first draft, and review and critique. KD: organization, execution of the study design, review, and critique. RB: execution of the study design, review, and critique. MK: conception, organization, of the study design, review, and critique. AE: conception, organization of the study design, review, critique, and final approval of the submitted version. All authors contributed to subsequent drafts and approved the submitted version.

## Conflict of Interest

LM has received honoraria from the International Association of Parkinsonism and Related Disorders (IAPRD) Society for social media and web support. MK is an employee of the CONICET and has received grant support from the Ministry of Science and Technology of Argentina and the Ministry of Health of Buenos Aires. AE has received grant support from the NIH and the Michael J Fox Foundation; personal compensation as a consult-ant/scientific advisory board member for Abbvie, Neuroderm, Neurocrine, Amneal, Adamas, Acadia, Acorda, Kyowa Kirin, Sunovion, Lundbeck, and USWorldMeds; publishing royalties from Lippincott Williams & Wilkins, Cambridge University Press, and Springer; and honoraria from USWorldMeds, Acadia, and Sunovion. He is cofounder of REGAIN Therapeutics, owner of a provisional patent on compositions and methods for treatment and/or prophylaxis of proteinopathies. The remaining authors declare that the research was conducted in the absence of any commercial or financial relationships that could be construed as a potential conflict of interest.

## Publisher's Note

All claims expressed in this article are solely those of the authors and do not necessarily represent those of their affiliated organizations, or those of the publisher, the editors and the reviewers. Any product that may be evaluated in this article, or claim that may be made by its manufacturer, is not guaranteed or endorsed by the publisher.

## Glossary

- **Read mapping** = Read mapping consists of the process of aligning the reads to a reference genome. The mapper uses as input a reference genome and a set of reads. The aim is to align each read in the set of reads on the specific reference genome.
- **Amplicon sequencing** = Amplicon Sequencing represents a targeted approach that enables researchers to analyze selected genetic variation(s) in specific genomic regions. The sequencing of PCR products (e.g., amplicons) allows variant identification and characterization. However, this approach is subject to inherent PCR biases.
- **CRISPR/Cas9-based amplification-free target enrichment method (also known as No-Amp targeted sequencing)** = CRISPR/Cas9 is a revolutionary gene editing tool of short-guide RNAs to target gene-modifying enzymes to specific DNA sequences. LRS is able to target genomic regions such as long repeats, without the need of PCR, e.g. avoiding PCR biases. This method has been called No-Amp targeted sequencing by Pac Bio. However, it can be used with both LRS platforms.
- **PCR bias** = It consists of artifacts introduced by PCR that skew the distribution of PCR products due to an unequal allelic amplification efficiency.
- **PCR stutter =** Stutters are common artifacts of amplification of the short-tandem repeat (STR) regions occurring when using polymerase chain reaction (PCR). They consist of strands of one or more motifs shorter or longer than the parental allele.
- **Haplotype phasing =** Haplotype phasing, or estimation in human genetics, refers to the process of statistical estimation of haplotypes from genotype data. The most common scenario arises when genotypes are collected at a set of polymorphic sites from a group of individuals. LRS allows for direct haplotype phasing, without statistical inference.
- **Satellite DNA** = DNA containing several tandem (e.g., not inverted) repeats of a given short basic repeating unit. Satellite DNA is located near the centromeres and telomeres of chromosomes (masked satellites).
