# Supplementary material for: Uncovering Essential Tremor Genetics: The Promise of Long-Read Sequencing
Source: Front Neurol. 2022 Mar 23;13:821189. doi: 10.3389/fneur.2022.821189 (PMC8983820; doi:10.3389/fneur.2022.821189)
Supplement: Supplementary file 1 [file Data_Sheet_1.docx]

**Supplementary Material**

**Supplementary Methods**

We conducted a systematic review from three data sources: Pubmed (Medline), Cochrane and Clinicaltrials.gov using the key terms (1) "sequence analysis, DNA," "high throughout nucleotide sequencing," "long read," "third generation," "single molecule real time," or "nanopore sequencing," and (2) "nervous system disorders" (complete list of search terms in **Supplementary Table**, below). The hedge for Humans was used in Pubmed, but no other restrictions were used. All categories of studies in clinicaltrials.gov were included (not yet recruiting, recruiting, enrolling, active, suspended, terminated, completed, withdrawn or unknown status). Articles in all languages and since databases’ inception were explored. The search was conducted on October 7^th^, 2021.

Articles about long-read sequencing and neurological diseases in adults were included. Studies on children or congenital conditions were excluded. **Supplementary Figure** shows the flow diagram of the systematic review. For Table 2 of the manuscript, we focused on studies on mutations uncovered only after long-read sequencing and excluded those where long-read sequencing did not add more information about mutations previously found by other genetic techniques. **Supplementary References** list those articles that used LRS to study mutations previously found by other genetic techniques and are not included in Table 2 of the manuscript.

# **Supplementary Table: Search terms**

| **Pubmed (MEDLINE)** | |
| --- | --- |
| 1 | **"long read"[Title/Abstract]** |
| 2 | **"third generation sequencing"[Title/Abstract]** |
| 3 | **"single molecule real time"[Title/Abstract]** |
| 4 | **"smrt sequencing"[Title/Abstract]** |
| 5 | **"nanopore sequencing"[MeSH Terms]** |
| 6 | **"nanopore sequencing"[Title/Abstract]** |
| 7 | **"pacbio"[Title/Abstract]** |
| 8 | **"oxford nanopore"[Title/Abstract]** |
| 9 | **"minion"[Title/Abstract]** |
| 10 | **"promethion"[Title/Abstract]** |
| 11 | **"gridion"[Title/Abstract]** |
| 12 | **"**SmidgION**"[Title/Abstract]** |
| 13 | **"nervous system diseases"[MeSH Terms]** |
| 14 | **"nerve tissue proteins"[MeSH Terms]** |
| 15 | **"c9orf72 protein"[MeSH Terms]** |
| 16 | **neuro*[Title/Abstract]** |
| 17 | **"nervous system"[Title/Abstract]** |
| 18 | **"tremor"[Title/Abstract]** |
| 19 | **"tremulous"[Title/Abstract]** |
| 20 | **"fragile x syndrome"[Title/Abstract]** |
| 21 | **epilep*[Title/Abstract]** |
| 22 | myoclon***[Title/Abstract]** |
| 23 | **ataxi*[Title/Abstract]** |
| 24 | **dystroph*[Title/Abstract]** |
| 25 | **parkinson*[Title/Abstract]** |
| 26 | **huntingt*[Title/Abstract]** |
| 27 | **"alzheimer"[Title/Abstract]** |
| 28 | dement***[Title/Abstract]** |
| 29 | **leukod*[Title/Abstract]** |
| 30 | **leukoe*[Title/Abstract]** |
| 31 | **"**neuronal intranuclear inclusion disease**"[Title/Abstract]** |
| 32 | **"**oculopharyngodistal myopathy**"[Title/Abstract]** |
| 33 | **"motor neuron disease"[Title/Abstract]** |
| 34 | "amyotrophic lateral sclerosis"**[Title/Abstract]** |
| 35 | dyston***[Title/Abstract]** |
| 36 | **"animals"[MeSH Terms]** |
| 37 | **"humans"[MeSH Terms]** |
| 38 | ({OR #1-#12}) AND ({OR #13-#35}) NOT (#36 NOT #37) |
| **Cochrane Central Register of Controlled Trials (CENTRAL)** | |
| 1 | [mh “sequence analysis, DNA”] |
| 2 | [mh “High-Throughput Nucleotide Sequencing”] |
| 3 | **[mh "nanopore sequencing"]** |
| 4 | (Long NEXT read*):ti,ab,kw |
| 5 | **("third generation" NEXT seq*):ti,ab,kw** |
| 6 | (“single molecule real time”):ti,ab,kw |
| 7 | (“smrt sequencing”):ti,ab,kw |
| 8 | (“nanopore sequencing”):ti,ab,kw |
| 9 | (pacbio):ti,ab,kw |
| 10 | (“oxford nanopore”):ti,ab,kw |
| 11 | (“gridion”):ti,ab,kw |
| 12 | [mh “nervous system diseases”] |
| 13 | [mh “nerve tissue proteins”] |
| 14 | [mh “c9orf72 protein”] |
| 15 | (neuro*):ti,ab,kw |
| 16 | (“nervous system”):ti,ab,kw |
| 17 | (tremor):ti,ab,kw |
| 18 | (tremulous):ti,ab,kw |
| 19 | (“fragile x syndrome”):ti,ab,kw |
| 20 | (epilep*):ti,ab,kw |
| 21 | (myoclon*):ti,ab,kw |
| 22 | (atax*):ti,ab,kw |
| 23 | (dystrop*):ti,ab,kw |
| 24 | (Parkinson*):ti,ab,kw |
| 25 | (huntingt*):ti,ab,kw |
| 26 | (dement*):ti,ab,kw |
| 27 | (Alzheimer*):ti,ab,kw |
| 28 | (leukod*):ti,ab,kw |
| 29 | (leukoe*):ti,ab,kw |
| 30 | (“neuronal intranuclear inclusion disease”):ti,ab,kw |
| 31 | (niid):ti,ab,kw |
| 32 | (“oculopharyngodistal myopathy”):ti,ab,kw |
| 33 | (“motor neuron disease”):ti,ab,kw |
| 34 | (“amyotrophic lateral sclerosis”):ti,ab,kw |
| 35 | (dyston*):ti,ab,kw |
| 36 | **[mh "Animals"]** |
| 37 | **[mh "Humans"]** |
| 38 | ({OR #1-#11}) AND ({OR #12-#35}) NOT (#36 NOT #37) |
| **ClinicalTrials.gov^a^** | |
| **Condition or disease:**  (Neurologic OR dystrophy OR dementia OR Epilepsy OR neural OR (nerve tissue protein))  **Other terms:**  ((long read) OR (High Throughput Nucleotide Sequencing) OR (sequence analysis DNA) OR (third AND generation AND sequencing) OR (oxford nanopore)) | |

^a^All terms entered in Pubmed and Cochrane were also included on ClinicalTrials.gov, but those that did not add new records were abandoned due to ClinicalTrials.gov’s character limits in the search terms.

**Supplementary Figure: Flow diagram**

Full-text articles assessed for eligibility
(n = **204**)

Records excluded
(n = **475**)

**679** Records screened

**679** Records after duplicates removed

## Identification

## Eligibility

## Screening

**677** records identified through database searching
(Pubmed: n= **207**; Cochrane Library: n= **69**; Clinicaltrials.gov: n= **401**)

Additional records identified through other sources^a^
(n = **2**)

Full-text articles excluded (n=**144**)

- Clinicaltrials.gov full records not about LRS (122)
- No LRS (15)
- Children or congenital (5)
- No patients reported (2)

Studies on mutations previously found by other genetic techniques
(n = **5**)^c^

Studies included in qualitative synthesis
(n = **60**)^b^

Studies about mutations uncovered by LRS
(n = **55**)^d^

^a^ References found in relevant reviews about LRS; ^b^ 59 peer-reviewed articles and 1 study found in clinicaltrials.gov; ^c^ Studies listed below in Supplementary References; ^d^ Studies presented in Table 2 of the manuscript.

LRS, long-read sequencing

**Supplementary References:**

Articles that used LRS to study mutations previously found by other genetic techniques and are not included in Table 2 of the manuscript:

Claassen D, Corey-Bloom J, Dorsey E, Edmondson M, Kostyk SK, LeDoux MS, Reilmann R, Diana Rosas H, Walker F, Wheelock V, Svrzikapa N, Longo KA, Goyal J, Hung J, Panzara MA. Neurol Genet Jun 2020, 6 (3) e430; doi: 10.1212/NXG.0000000000000430

De Roeck A, Van den Bossche T, van der Zee J, Verheijen J, De Coster W, Van Dongen J, Dillen L, Baradaran-Heravi Y, Heeman B, Sanchez-Valle R, Lladó A, Nacmias B, Sorbi S, Gelpi E, Grau-Rivera O, Gómez-Tortosa E, Pastor P, Ortega-Cubero S, Pastor MA, Graff C, Thonberg H, Benussi L, Ghidoni R, Binetti G, de Mendonça A, Martins M, Borroni B, Padovani A, Almeida MR, Santana I, Diehl-Schmid J, Alexopoulos P, Clarimon J, Lleó A, Fortea J, Tsolaki M, Koutroumani M, Matěj R, Rohan Z, De Deyn P, Engelborghs S, Cras P, Van Broeckhoven C, Sleegers K; European Early-Onset Dementia (EU EOD) consortium. Deleterious ABCA7 mutations and transcript rescue mechanisms in early onset Alzheimer's disease. Acta Neuropathol. 2017 Sep;134(3):475-487. doi: 10.1007/s00401-017-1714-x. Epub 2017 Apr 27. PMID: 28447221; PMCID: PMC5563332.

Höijer I, Tsai YC, Clark TA, Kotturi P, Dahl N, Stattin EL, Bondeson ML, Feuk L, Gyllensten U, Ameur A. Detailed analysis of HTT repeat elements in human blood using targeted amplification-free long-read sequencing. Hum Mutat. 2018 Sep;39(9):1262-1272. doi: 10.1002/humu.23580. Epub 2018 Jul 12. PMID: 29932473; PMCID: PMC6175010.

Perrone F, Bjerke M, Hens E, Sieben A, Timmers M, De Roeck A, Vandenberghe R, Sleegers K, Martin JJ, De Deyn PP, Engelborghs S, van der Zee J, Van Broeckhoven C, Cacace R; BELNEU Consortium. Amyloid-β1-43 cerebrospinal fluid levels and the interpretation of APP, PSEN1 and PSEN2 mutations. Alzheimers Res Ther. 2020 Sep 11;12(1):108. doi: 10.1186/s13195-020-00676-5. PMID: 32917274; PMCID: PMC7488767.

Svrzikapa N, Longo KA, Prasad N, Boyanapalli R, Brown JM, Dorset D, Yourstone S, Powers J, Levy SE, Morris AJ, Vargeese C, Goyal J. Investigational Assay for Haplotype Phasing of the Huntingtin Gene. Mol Ther Methods Clin Dev. 2020 Sep 11;19:162-173. doi: 10.1016/j.omtm.2020.09.003. PMID: 33209959; PMCID: PMC7648085.
